# Doubling the diagnostic rate of at-risk metabolic dysfunction-associated steatohepatitis—leave no one behind

**DOI:** 10.1016/j.lanepe.2025.101572

**Published:** 2025-12-19

**Authors:** Elamin Abdelgadir, Fatheya Alawadi, Fauzia Rashid, Alaaeldin Bashir, Jörn M. Schattenberg

**Affiliations:** a Endocrinology Department, Mohamed Bin Rashid University of Medicine and Health Sciences, Dubai, United Arab Emirates; b Endocrine Department, Dubai Hospital, Dubai, Dubai Health, United Arab Emirates; c Department of Internal Medicine II, Saarland University Medical Centre, Homburg, Germany

We read with great interest the “call to arms” by Lazarus and colleagues to double the diagnosis rate of at-risk metabolic dysfunction-associated steatohepatitis (MASH) by 2027 in France, Germany, the UK, and the USA.^1^ Sensibly, the authors advocate for a pragmatic risk-based approach to identify those with ‘at-risk’ MASH (MASH with F2 or F3 fibrosis) by enriching for the population with metabolic dysfunction-associated steatotic liver disease (MASLD) and MASH (individuals with T2D, obesity and one or more cardiometabolic risk factor(s), and elevated liver enzymes) and then active case finding through non-invasive tests like FIB-4, in line with 2024 EASL-EASD-EASO clinical practice guidelines.^2^ The hope is to channel individuals into optimized care pathways for the early detection and prevention of fibrosis progression into cirrhosis and hepatocellular carcinoma.

While we acknowledge that the strategies proposed by the authors may be generalizable, we are concerned that the regional bias in the paper, which received extensive media coverage, risks exacerbating regional health inequity by framing MASH as a “Western” problem. Indeed, our nationally representative data from the Middle East^3^ highlight the scale of the problem locally (see Table 1): half (n = 46,412, 49.0%) of the population would screen positive for being at risk of MASLD and, of those, about a fifth (21.7%) are at moderate-high risk of advanced fibrosis. Although it is tempting to attribute this high prevalence to the obesity epidemic, we also note that 15.0% of individuals without MASLD risk factors, including obesity, are at moderate-high risk of fibrosis. It will be important to identify these individuals too, as “lean” MASLD is common and associated with worse outcomes.^4^^,^^5^

**Table 1:** The prevalence of MASLD-related risk factors and association with FIB-4 status in a cross-sectional, multicenter, population-based study of all adults aged >18 years attending the Dubai Academic Health Corporation (DAHC) between January 2018 and August 2023.^3^

	MASLD risk factors absent (N = 48,342)^c^	MASLD risk factors present (N = 46,412)^c^	Total (N = 94,754)	p value
**Age**				< 0.001^a^
Mean (SD)	41.9 (15.5)	53.6 (15.5)	47.6 (16.5)	
**Age category**				< 0.001^b^
19–39	24697.0 (51.1%)	9364.0 (20.2%)	34061.0 (35.9%)	
40–64	19013.0 (39.3%)	25329.0 (54.6%)	44342.0 (46.8%)	
≥65	4632.0 (9.6%)	11719.0 (25.2%)	16351.0 (17.3%)	
**Sex**				< 0.001^b^
Female	32388.0 (67.0%)	22765.0 (49.0%)	55153.0 (58.2%)	
Male	15954.0 (33.0%)	23647.0 (51.0%)	39601.0 (41.8%)	
**FIB-4**^d^				< 0.001^b^
Low risk	41104.0 (85.0%)	36359.0 (78.3%)	77463.0 (81.8%)	
Moderate to high risk	7238.0 (15.0%)	10053.0 (21.7%)	17291.0 (18.2%)	

a Student *t-*test.

b Pearson's chi-squared test.

c MASLD risk factors were defined as the presence of any of: (i) type 2 diabetes, defined according to ICD-10 codes; (ii) obesity (defined according to WHO standards as a BMI ≥30.0–34.9 kg/m^2^ for individuals not from Southeast Asia and ≥25.0 kg/m^2^ for those from Southeast Asia^3^) together with ≥1 cardiometabolic risk factor (prediabetes defined as an HbA1c 5-7-6.4, hyperlipidemia defined according to hyperglycemia, high triglyceride levels ≥1.7 mmol/L, hypertension diagnosed according to ICD-10 codes and consistent with EASL-EASD-EASO clinical practice guidelines^2^); and (iii) ALT >33 U/L in males and >25 U/L in females.

d FIB-4 was calculated as: age (years) × aspartate aminotransferase (AST [U/L])/(platelets [10^9^/L] × alanine aminotransferase [ALT (U/L)]^1/2^), with AST and ALT measured on the same day and platelets within ±30 days and was categorized as low (<1.30 in individuals <65 years, <2.0 in individuals ≥65 year), moderate (1.30–2.67 or 2.0–2.67, depending on age), or high (>2.67) risk of advanced fibrosis using cut-offs previously shown to be associated with advanced fibrosis and in alignment with clinical guidelines.

The expenses of testing are also concern, as current guidelines support a two-tier approach with additional tests being required in the FIB-4 >1.3 population. As an alternative approach, investment in preventative strategies–even in the absence of testing–and education on liver healthy diets will be an important aspect of maintaining population health.

We value the “knowledge, tools, and opportunity to end this public health threat” proposed by the authors, but we need to make sure that no individual in any part of the world is left behind.

## Contributors

Conceptualization: EA, FR, AB, FA; Formal analysis: EA, FR, AB, FA; Data curation: EA, FR, AB; Writing–Original Draft: EA, FR, AB, JMS, FA; Writing–Review & Editing: EA, FR, AB, JMS, FA.

## Declaration of interests

EA received an unrestricted research grant from Novo Nordisk-UAE and received honoraria for lectures and presentations and support for attending meetings and travel from Boehringer Ingelheim, Bayer, AstraZeneca, and Novo Nordisk.

FA has received honoraria for lectures and presentations from Bayer, Novo Nordisk, and Boehringer Ingelheim and support for attending meetings and travel from Boehringer Ingelheim, AstraZeneca, and Novo Nordisk.

FR has received honoraria for lectures and presentations and support for attending meetings and travel from Boehringer Ingelheim, AstraZeneca, and Novo Nordisk.

AB has received honoraria for lectures and presentations from MSD, Boehringer Ingelheim, Bayer, and Novo Nordisk and support for attending meetings and travel from MSD, Boehringer Ingelheim, AstraZeneca, and Novo Nordisk.

JMS has received consulting fees from Akero, Alentis, Alexion, Altimmune, Astra Zeneca, 89Bio, Bionorica, Boehringer Ingelheim, Boston Pharmaceuticals, Gilead Sciences, GSK, HistoIndex, Ipsen, Inventiva Pharma, Madrigal Pharmaceuticals, PRO.MED.CS Praha a.s., and Kríya Therapeutics; honoraria for lectures and presentations from AbbVie, Boehringer Ingelheim, Gilead Sciences, Ipsen, Lilly, Novo Nordisk, Madrigal Pharmaceuticals; and has stock options in Hepta Bio.
